# Preliminary Evaluation of a Novel Thermoplastic Mask System with Intra-fraction Motion Monitoring for Future Use with Image-Guided Gamma Knife

**DOI:** 10.7759/cureus.531

**Published:** 2016-03-13

**Authors:** Winnie Li, Gregory Bootsma, Oscar Von Schultz, Per Carlsson, Normand Laperriere, Barbara-Ann Millar, David Jaffray, Caroline Chung

**Affiliations:** 1 Department of Radiation Oncology, Princess Margaret Cancer Centre, Toronto, ON; 2 Radiation Medicine Program, Princess Margaret Cancer Centre, Toronto, ON / University Health Network, Toronto, ON; 3 Elekta Research & Development, Elekta Instruments AB, Stockholm, Sweden

**Keywords:** immobilization, intra-fraction motion, cone beam, stereotatic radiosurgery, optical tracking

## Abstract

**Objectives:**

A non-invasive immobilization system consisting of a thermoplastic mask with image-guidance using cone-beam CT (CBCT) and infrared (IR) tracking has been developed to ensure minimal inter- and intra-fractional movement during Gamma Knife radiosurgery. Prior to clinical use for patients on a Gamma Knife, this study clinically evaluates the accuracy and stability of this novel immobilization system with image-guidance in patients treated with standard fractionated radiation therapy on a linear accelerator.

**Materials & methods:**

This prospective cohort study evaluated adult patients planned for fractionated brain radiotherapy. Patients were immobilized with a thermoplastic mask (with the nose cut out) and customized head cushion. A reflective marker was placed on the patient’s nose tip and tracked with a stereoscopic IR camera throughout treatment. For each fraction, a pre-treatment, verification (after any translational correction for inter-fraction set-up variation), and post-treatment CBCT was acquired to evaluate inter- and intra-fraction movement of the target and nose. Intra-fraction motion of the nose tip measured on CBCT and IR tracking were compared.

**Results:**

Corresponding data from 123 CBCT and IR datasets from six patients are summarized. The mean ± standard deviation (SD) intra-fraction motion of the nose tip was 0.41±0.36 mm based on pre- and post-treatment CBCT data compared with 0.56±0.51 mm using IR tracking. The maximum intra-fraction motion of the nose tip was 1.7 mm using CBCT and 3.2 mm using IR tracking. The mean ± SD intra-fraction motion of the target was 0.34±0.25 mm, and the maximum intra-fraction motion was 1.5 mm.

Conclusions: This initial clinical evaluation of the thermoplastic mask immobilization system using both IR tracking and CBCT demonstrate that mean intra-fraction motion of the nose and target is small. The presence of isolated measures of larger intra-fraction motion supports the need for image-guidance and intra-fraction motion management when using this mask-based immobilization system for radiosurgery.

## Introduction

Historically, intracranial radiosurgery has involved single large dose treatments using stereotactic localization and immobilization with an invasive head-frame [1]. The first radiosurgery treatments were performed on a Gamma Knife in the early 1970s [2]. Since that time, advances in the Gamma Knife model design have introduced a larger internal cavity to reduce the risk of collisions while allowing for a larger treatable volume within the brain, orbit, and base of skull [3]. The re-engineered design of the couch and sources has enabled treatment of larger, more extensive target volumes with relatively little increase in overall treatment time. The larger internal cavity of the Leksell Gamma Knife® Perfexion™ unit (Elekta Instrument, AB, Sweden) also introduced the potential for frameless radiosurgery and non-invasive, mask-based fractionated treatment while maintaining high conformality and accuracy.

The eXtend™ Relocatable Head Frame (RHF), a modified version of the commercially available Headfix frame (Medical Intelligence, Schwabmunchen, Germany) [4-5], was developed for use on the Perfexion Gamma Knife unit for fractionated radiosurgery. Immobilization with the RHF showed equivalent or better accuracy than that obtained with standard care Gill-Thomas-Cosman relocatable frames [6-8]. The positioning and immobilization accuracy of the RHF was validated for fractionated stereotactic linear accelerator-based radiotherapy for intracranial, orbital, and base-of-skull lesions in patients with a full set of teeth [9], with similar positioning uncertainties reported on the Gamma Knife [10]. However, the RHF system has not been evaluated in edentulous patients, and vacuum fixation to the patient’s hard palate has resulted in challenges with both patient fit and tolerance with this system. Anecdotally from our institution’s experience, a consistent vacuum seal was not achievable in some patients and the vacuum system lead to irritation and minor bleeding of the hard palate in others.

With the addition of cone-beam computed tomography (CBCT), Image-Guided Perfexion (IGP) allows quantification of patient positional accuracy prior to Gamma Knife radiosurgery. In order to overcome challenges with the RHF and offer non-invasive immobilization for both dentulous and edentulous patients, a novel immobilization system consisting of a thermoplastic mask with infrared (IR) tracking of a patient nose marker as the intra-fraction motion management (IFMM) system in addition to CBCT image-guidance has been developed for the Gamma Knife unit. The primary objective of this study was to quantify the accuracy of the thermoplastic mask system with IFMM in a sample of intracranial patients undergoing standard care fractionated linear accelerator-based radiotherapy, serving as a baseline for fractionated Gamma Knife radiosurgery. The secondary objectives were to assess the inter- and intra-fraction motion in the mask system, and measure target motion relative to patient motion with CBCT and IR optical tracking.

## Materials and methods

### Eligibility criteria

This prospective study included adult patients (≥18 years old) with intracranial tumors planned for fractionated linear accelerator-based radiotherapy with an Eastern Cooperative Oncology Group performance status of 0–2. Informed consent was obtained.

### Treatment planning

Volumetric imaging was acquired for treatment planning purposes, including gadolinium-enhanced axial T1-weighted magnetic resonance (MR) images with 1 mm slice thickness, T2-weighted fluid attenuated inversion recovery (FLAIR) MR images, and CT simulation (2 mm slice thickness). Helical CT scans were acquired with 120 kV and 300 mAs, reconstructed at 0.5x0.5x2.0 mm voxels. The gross tumor volume (GTV) and clinical target volume (CTV) were defined by the treating radiation oncologist, with a 3–5 mm planning target volume (PTV) margin added to the CTV as per standard clinical practice for intracranial patients treated in the standard mask with intensity modulated radiation therapy (IMRT) or volumetric modulated arc therapy (VMAT) along with CBCT image-guidance. All treatment plans were generated using the same treatment planning system (Pinnacle v9.6, Philips, Milpitas, CA), and treated with either step-and-shoot IMRT or VMAT. The fractionation schemes included 2000 cGy/5, 4005 cGy/15 and 6000 cGy/30.

### Immobilization system

**Figure 1:**
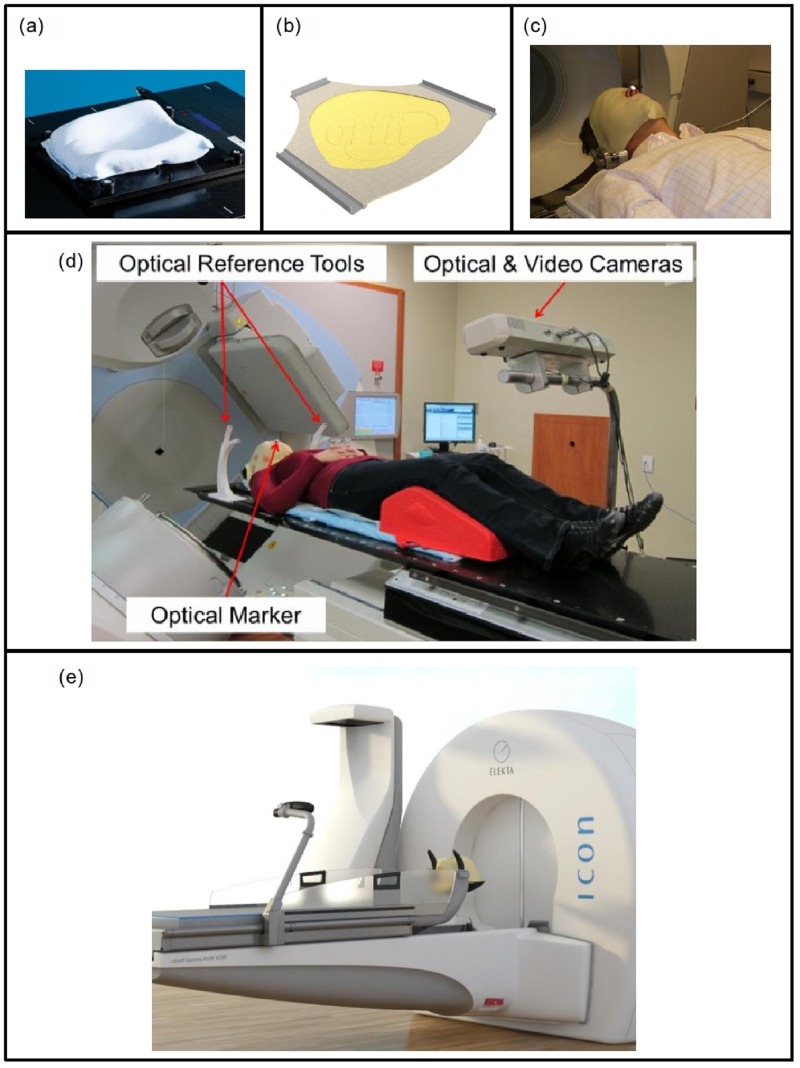
The thermoplastic mask system (a) A resin-filled cushion and (b) thermoplastic mask was customized for each patient, with the portion of the mask covering the patient’s nose removed. (c) During treatment, an optical marker was placed on the patient’s nose to monitor intra-fraction motion. (d) Representative image of patient setup, immobilized on the thermoplastic mask system. The coordinates of the reflective marker on the patient’s nose were stereoscopically acquired by the camera, and are used by the software to continuously track and calculate the 3D position of the marker. Displacements between the markers on the patient’s nose and the reference tool were used to determine intra-fraction motion. (e) The mask system on the Perfexion Icon™ platform.

The thermoplastic mask system consisted of a customized resin-filled neck rest (AccuForm™, Civco Medical Solutions, Kalona, IA) and a 3-point thermoplastic mask (Orfit® Industries, Belgium) (Figures 1A, 1B). The nose portion of the mask was cut out to allow placement of a single reflective optical marker on the patient’s nose (anterior columella) as this represents a stable anatomical reference point for intra-fraction motion tracking, visible inside the radiation unit (Figure 1C). The mask cut-out was created to ensure the lateral edges of the nose (cartilage) were not in contact with the mask to reduce interference and noise of nose versus target (cranium) motion.

### Clinical workflow and CBCT image acquisition

Patients were treated on a linear accelerator (Elekta Agility®, Crawley, UK) equipped with an orthogonal mounted X-ray tube and amorphous silicon flat panel detector (CBCT system). CBCT scans were acquired over two minutes with a gantry rotation of 360°, a frame rate of 5.5 Hz, and an estimated maximum dose (on phantom) of 6 mGy at a 2-cm depth [11]. All volumetric CBCT datasets were reconstructed with 400x400x256 voxels at a 1 mm isotropic voxel size.

**Figure 2:**
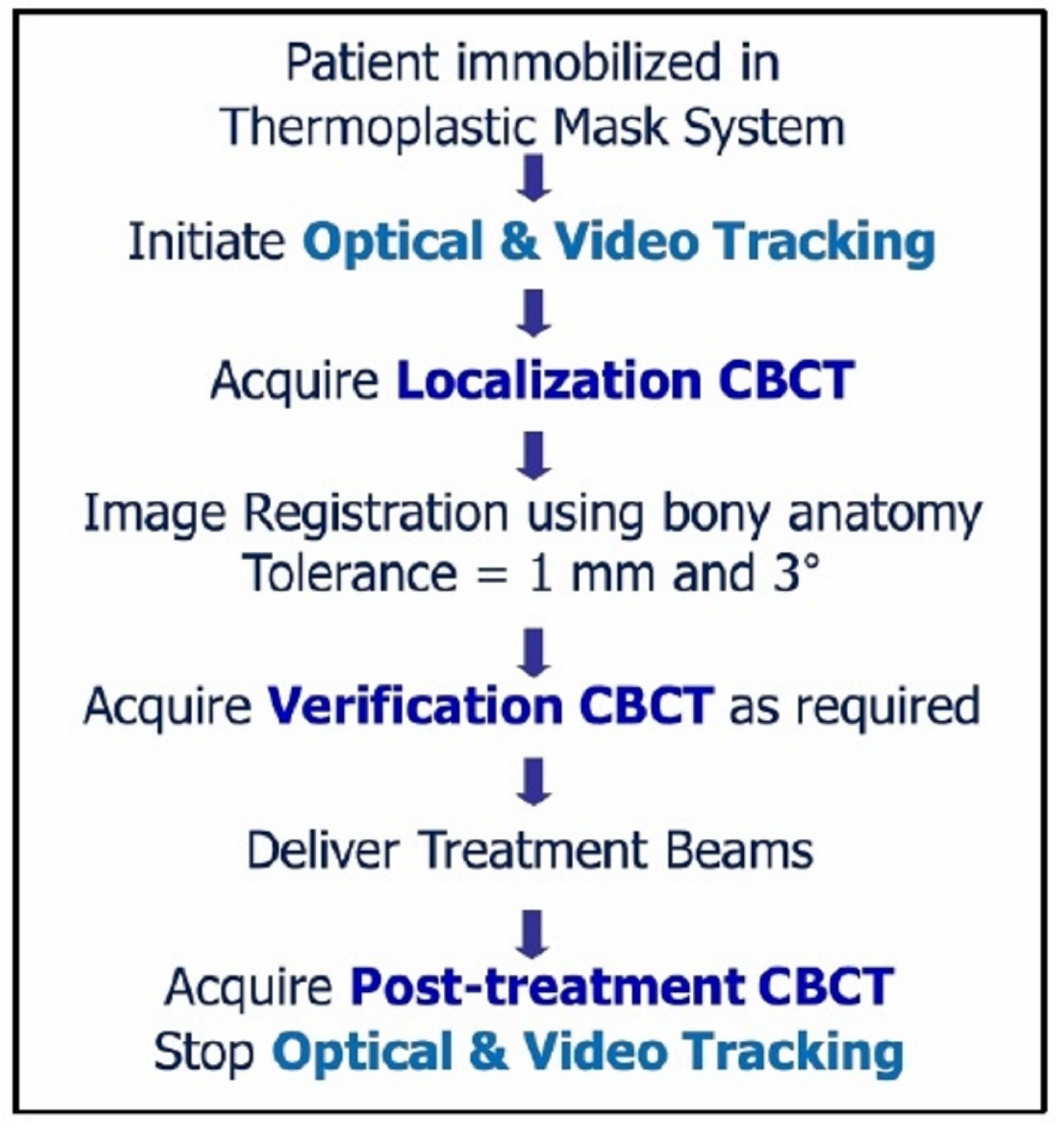
Schema of the thermoplastic mask system image-guidance workflow

The clinical workflow for target localization and verification included CBCT imaging and registration (see Figure 2). After patient immobilization and alignment to the setup point on the mask, an online localization CBCT was acquired prior to treatment and analyzed through the imaging software (X-ray Volumetric Imaging, v4.5, Elekta, Stockholm), with tolerances for couch adjustment following standard clinical practice (i.e. 1 mm).

**Figure 3:**
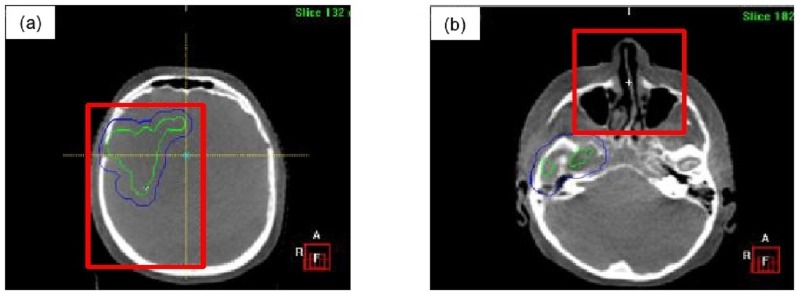
Example regions of interest (red outline) for automatic CBCT image registration. Representative coronal images for: a) target, b) nose.

Figure 3A illustrates an example of a region of interest (i.e. bounding clipbox) used for automatic rigid image registration of bony anatomy through mutual information to assess translational and rotational offsets. Measured translational discrepancies were corrected by the treatment couch and rotational offsets were monitored to ensure clinical tolerance was maintained (i.e. 3°), initiating patient re-positioning if threshold is exceeded. After any image-guided couch adjustments to resolve translational discrepancies, a verification CBCT was performed to assess residual setup errors prior to treatment delivery. At the end of the treatment, a post-treatment CBCT was acquired to assess intra-fraction motion.

### Intra-fraction monitoring system

Passive optical tracking was used to assess immobilization accuracy of the mask system by monitoring intra-fraction motion. The optical monitoring system (Figure 1D) consists of customized software, a camera mount and an IR emitting stereoscopic camera (Polaris Spectra®, Northern Digital Inc., Waterloo, Canada) [12]. The use of a stereoscopic camera enables depth information to be obtained from the optical marker. Two optical tracking reference tools (curved white devices, Figure 1D) were fixed to the treatment couch to provide a stable frame of reference. This mirrors the setup of the immobilization system on the Gamma Knife Icon™ platform (see Figure 1E).The optical tracking software was designed to record the 3D displacement of the patient marker relative to the optical reference tools to ensure the patient has not moved outside of set thresholds. The optical camera enabled marker tracking at a maximum rate of 60 Hz with a manufacturer reported accuracy of 0.25 mm.

In addition to IR optical tracking, a video camera was affixed to the optical camera to facilitate video recording during each treatment session. The recorded video data was used to correlate any intra-fraction motion measured through either optical tracking or CBCT imaging, and to investigate the cause of any large motions detected on optical tracking or CBCT imaging.

### Study data collection & analysis

Localization, verification, and post-treatment CBCT images were retrospectively registered by a single observer (W.L.) using both the clinically defined region of interest covering target (Figure 3A) and the nose region (Figure 3B) through two separate co-registrations. Similar to the clinical workflow, automatic rigid image registration of the bony anatomy within the defined regions of interest was performed through mutual information to assess translational and rotational discrepancies. The mean and SD for the two separate co-registrations were calculated for each patient, and subsequently the group mean (M), population systematic (Σ) and random (σ) errors were determined. The random error was calculated from the root mean square of all SDs and the systematic error was calculated from the SD of all means.

Intra-fraction motion for each treatment fraction based on CBCT was quantified through subtraction of discrepancies measured on the post-treatment CBCT from either the localization (i.e. images registered within clinical threshold) or verification (i.e. cases where a couch adjustment was required) CBCTs. A three-dimensional vector magnitude was calculated from the intra-fraction displacements in the left-right (L-R), cranial-caudal (C-C), and anterior-posterior (A-P) directions using both the target and nose regions of interest. The intra-fraction displacement measured from the target and nose regions of interest on the CBCTs were compared to the vector displacements measured from passive optical tracking.

The 3D optical tracking information transmitted by the camera was pro­cessed by a specialized software interface written in object oriented C++ using open source libraries for 3D visualiza­tion (Coin3D and SIM Voleon). The software recorded the 3D localization information of the nose marker and 2D video from the cameras at a rate of 16 Hz. The optical tracking and video data was post-processed using a commercial software package (MATLAB v8.1, The MathWorks Inc., Natick, MA) to determine the vector displacement of the nose marker shift from the pre-treatment CBCT to the post-treatment CBCT. The time of the CBCT scans was automatically determined through a novel image processing algorithm run on the synced video data. The nose vector displacement of the optical data was computed by determining the difference between the mean position of the marker during the pre-treatment scan (i.e. baseline) and the mean position of the marker during the post-treatment scan.

## Results

Seven patients were accrued to the study, but one patient withdrew prior to treatment due to unexpected rapid clinical deterioration. A total of 140 fractions were evaluated in six patients with 140 localization, 122 verification, and 129 post-treatment CBCTs. Post-treatment CBCTs were not captured on all treatment fractions due to either patient- or hardware-related factors (i.e. patient could not tolerate treatment position after radiation delivery, imaging panel calibration issues). The average time between pre- and post-treatment CBCT acquisition was 7 minutes 38 seconds (range 4 minutes 36 seconds–13 minutes 41 seconds). Patient and treatment characteristics are summarized in Table 1.

**Table 1:** Patient characteristics

Patient #	Age, Gender	Race	Tumor Location	# Fractions	Mean Treatment Time (Per Fraction)
1	41 yo F	Asian	Central	5	13:54 minutes
2	70 yo F	Caucasian	Anterior right	30	08:08 minutes
3	Withdrew prior to any treatment due to unexpected rapid clinical deterioration				
4	58 yo M	Caucasian	Posterior left	30	08:55 minutes
5	55 yo M	Caucasian	Posterior midline	15	12:26 minutes
6	40 yo F	Caucasian	Posterior midline	30	09:31 minutes
7	64 yo M	Caucasian	Anterior midline	30	14:46 minutes

### Inter-fraction motion

Table 2 summarizes the inter-fraction motion measured on localization, verification, and post-treatment CBCTs. Both systematic and random errors in all translational axes were reduced with image guidance. Larger systematic and random errors were observed with the nose region of interest image registration.

**Table 2:** Summary of systematic and random positional errors stratified by region of interest used for CBCT image registration

	Target			Nose		
	LR (mm)	CC (mm)	AP (mm)	LR (mm)	CC (mm)	AP (mm)
Localization CBCT						
Group Mean	-0.54	0.06	-1.24	-0.83	-0.33	-1.80
Systematic Error	0.47	1.18	0.50	0.52	1.74	0.28
Random Error	0.78	1.08	0.53	0.88	1.29	0.52
Verification CBCT						
Group Mean	0.04	-0.18	-0.02	-0.31	-0.53	-0.63
Systematic Error	0.16	0.14	0.17	0.47	0.81	0.68
Random Error	0.32	0.34	0.32	0.46	0.65	1.09
Post-Treatment CBCT						
Group Mean	0.04	-0.06	-0.19	-0.33	-0.36	-0.68
Systematic Error	0.25	0.18	0.22	0.53	0.70	0.48
Random Error	0.38	0.43	0.40	0.48	0.58	0.47

### Intra-fraction motion - CBCT

Table 3 summarizes the intra-fraction motion measured on CBCT and optical tracking. Using the target region of interest for image registration on CBCTs, the mean ± SD of measured intra-fraction motion was 0.05±0.22 mm, 0.05±0.30 mm, and -0.09±0.19 mm in the L-R, C-C, and A-P directions, respectively. Similarly, the mean ± SD of measured intra-fraction motion using the nose region of interest for image registration on CBCT data was 0.01±0.33 mm, 0.07±0.38 mm, and -0.07±0.18 mm in the L-R, C-C, and A-P directions, respectively. The vector measurement using the target region of interest was 0.34±0.25 mm, while the nose region of interest measurements yields a vector of 0.41±0.36 mm.

**Table 3:** Summary of intra-fraction motion based on CBCT registrations using both the target and nose regions of interest, and infrared tracking of the nose marker

	Target - CBCT						
	LR (mm)	CC (mm)	AP (mm)	Vector (mm)	Pitch (°)	Roll (°)	Yaw (°)
Mean	0.05	0.05	-0.09	0.34	0.02	-0.01	0.11
SD	0.22	0.30	0.19	0.25	0.45	0.25	0.33
Maximum	0.80	0.90	0.40	1.52	1.3	1.1	1.5
Minimum	-0.50	-1.20	-1.00		-2	-1	-0.8
	Nose - CBCT						
	LR (mm)	CC (mm)	AP (mm)	Vector (mm)	Pitch (°)	Roll (°)	Yaw (°)
Mean	0.04	0.09	-0.07	0.37	-0.03	0.01	0.11
SD	0.24	0.34	0.18	0.28	0.40	0.24	0.31
Maximum	0.9	1.5	0.4	1.72	1	1.3	1.5
Minimum	-0.5	-1.4	-0.9		-1.9	-0.5	-0.6
	Nose - Optical						
	LR (mm)	CC (mm)	AP (mm)	Vector (mm)			
Mean	-0.06	-0.01	-0.17	0.56			
SD	0.27	0.28	0.63	0.51			
Maximum	0.87	2.73	0.83	3.16			
Minimum	-1.28	-2.91	-0.90				

### Intra-fraction motion – optical tracking

IR optical and video tracking data was collected on 133 treatments. IR tracking of the passive nose optical marker resulted in a mean±SD intra-fraction motion of -0.06±0.27 mm, -0.17±0.63 mm, and -0.01±0.28 mm in the L-R, C-C, and A-P directions, respectively. The vector magnitude of 0.56±0.51 mm was larger than measured using CBCT data.

In the LR direction, 96% (128/133) of intra-fraction motion was within ±0.5 mm and 100% within ±1.0 mm. In the AP direction, 94.7% (126/133) of fractions were within ±0.5 mm, with all fractions maintained within ±1.0 mm. The largest motion was recorded in the CC axes, where 86.5% (115/133) of fractions were within ±0.5 mm, 95.5% (127/133) were within ±1.0 mm and 98.5% (131/133) were within ±2.0 mm. The range of intra-fraction motion was largest in the CC direction, with a minimum and maximum measured value of -2.9 mm and 2.7 mm, respectively. Upon investigation of these two fractions with larger intra-fraction motion, video footage of one indicated the patient had a sudden gross movement on the bed that corresponded with the detected large motion using IR and for the second patient, a gradual drift of the nose position was observed.

### Correlation

A total of 123 corresponding CBCT and optical datasets were available for six patients. Larger correlation was found between the vector intra-fraction motion of CBCT-target and CBCT-nose (r=0.828, range 0.23–0.97), while smaller correlation was observed between CBCT-nose and optical-nose (r=0.286, range 0.08–0.78). A larger magnitude of motion per fraction was consistently observed on optical tracking than CBCT, indicating its increased sensitivity to motion.

## Discussion

This prospective cohort study aimed to gather preliminary clinical measurements of the performance and accuracy of a thermoplastic mask system with IR tracking and CBCT image guidance for eventual use on a frameless radiosurgery Gamma Knife unit. Based on CBCT image registration results, the mask system reported intra-fraction motion (mean±SD) of 0.34±0.25 mm for the target (max 1.5 mm) and 0.41±0.36 mm for the nose (max 1.7 mm), similar to the clinically available RHF results of 0.4±0.3 mm [9]. As comparable accuracy was found between the devices, this supports the use of the thermoplastic mask system as an immobilization device for fractionated radiosurgery on the Gamma Knife unit when used with CBCT image guidance and intra-fraction motion management. A prototype CBCT system has previously been developed and evaluated in-house [13]; the commercial release of this image-guidance technology will enable clinical use of this immobilization system.

Several studies using bite block-based immobilization systems for radiosurgery have been evaluated through either IR tracking or CBCT imaging. When the bite block was used alone, the mean intra-fraction motion detected with IR tracking was reported to be less than 0.4 mm [14-15]. Other studies have evaluated immobilization systems composed of a bite block in conjunction with a mask through CBCT: Masi et al. reported intra-fraction motion of 0.2±0.55 mm, 0.1±0.61 mm, and 0.3±0.55 mm in the LR, CC, and AP axes, respectively [16];  Tryggestad et al. reported mean±SD intra-fraction motion of 0.71±0.8 mm [17]. In our study, the mean±SD intra-fraction motion measured based on passive IR tracking of the nose tip for a 3-point thermoplastic mask and custom headrest system without the use of a bite block was 0.56±0.51 mm, comparable to these other frameless immobilization devices in clinical use for radiosurgery.

As we used both IR tracking and CBCT image-guidance, intra-fraction motion measured through IR tracking of the patients’ nose tip was compared and found consistently greater than CBCT-based movement of the nose tip and treated target. This finding suggests that movement of the nose-tip measured by IR tracking is sensitive. Therefore monitoring and limiting the movement of the nose tip using real-time IR tracking would allow interruption of the treatment in order to ensure the target remains within a clinically acceptable threshold for intra-fraction motion. As this threshold value can be adjusted to the clinical situation, this immobilization system would provide flexibility in the accuracy with which targets are treated, considering the specific target and surrounding critical structures involved, as well as efficiency in workflow. The clinical benefit of very small thresholds for movement would need to be off-set by the efficiency of treatment delivery as setting very small thresholds would ensure higher accuracy, but may result in multiple pauses in treatment.

As this initial evaluation of the thermoplastic mask system was performed on patients receiving standard fractionated radiotherapy using a linear accelerator, the treatment times were generally shorter than for single fraction Gamma Knife radiosurgery. Other variables, such as differences in couch construction between a linear accelerator and the Gamma Knife, may also affect the reliability and accuracy of the thermoplastic immobilization system. Therefore this initial study only provides promising preliminary data to guide the use of the thermoplastic mask-based immobilization system with IFMM on Gamma Knife. Further clinical validation studies are planned to investigate the intra-fraction motion in patients treated with longer treatment times on the Gamma Knife. These subsequent studies will help determine appropriate thresholds for the IFMM system to maximize treatment accuracy and efficient workflow for mask-based treatments.

It is important to note that components of the thermoplastic mask system were shaped to patient specific anatomy with both the neck cushion and mask formed according to the individual patient anatomy at simulation. To ensure reproducibility, staff education and training is required to encourage consistent manufacture of the immobilization device for optimal effectiveness. Specifically, as the rigidity of the mask is reduced with the nose cutout, careful molding of the bridge of the patient’s nose is crucial to maintain immobilization. Additionally, the customized neck cushion activates upon contact with water, giving staff a limited window of time before the device hardens permanently. For our small subset of patients in this study, the same research personnel (W.L.) was present for the consistent manufacture of the immobilization system.

To facilitate fractionated Gamma Knife practices in the frameless immobilization system, the inclusion of data-driven PTV margin expansion on treated targets in the clinical workflow would be logical. Current PTV margin formulas in the literature are aimed at longer conventional fractionation schedules and linear-accelerator-based geometries [18]. We are currently exploring strategies to derive patient-specific anisotropic PTV margins for intracranial SRT to account for inter- and intra-fraction motion on the Gamma Knife [19]. With this IFMM system on the Gamma Knife, the PTV margin would be directly related to the thresholds set for the treatment, as these thresholds will directly impact the allowed motion of the target during treatment.

## Conclusions

This initial clinical evaluation of the immobilization accuracy of an image-guided thermoplastic mask system intended for use on Gamma Knife using IR tracking and CBCT demonstrated mean inter- and intra-fraction movements of < 1 mm. Target motion (measured on CBCT) was generally smaller (max 1.5 mm) than patient (nose/head) motion detected on CBCT (max 1.7 mm) and IR tracking (max 3.2 mm), suggesting image-guidance and intra-fraction motion management ensures accurate targeting on IGP.
